# Doxycycline modulates VEGF-A expression: Failure of doxycycline-inducible lentivirus shRNA vector to knockdown VEGF-A expression in transgenic mice

**DOI:** 10.1371/journal.pone.0190981

**Published:** 2018-01-19

**Authors:** Mari Merentie, Riina Rissanen, Line Lottonen-Raikaslehto, Jenni Huusko, Erika Gurzeler, Mikko P. Turunen, Lari Holappa, Petri Mäkinen, Seppo Ylä-Herttuala

**Affiliations:** 1 A. I. Virtanen Institute for Molecular Sciences, Faculty of Health Sciences, University of Eastern Finland, Kuopio, Finland; 2 Gene Therapy Unit, Kuopio University Hospital, Kuopio, Finland; 3 Heart Center, Kuopio University Hospital, Kuopio, Finland; Virginia Commonwealth University, UNITED STATES

## Abstract

Vascular endothelial growth factor-A (VEGF-A) is the master regulator of angiogenesis, vascular permeability and growth. However, its role in mature blood vessels is still not well understood. To better understand the role of VEGF-A in the adult vasculature, we generated a VEGF-A knockdown mouse model carrying a doxycycline (dox)-regulatable short hairpin RNA (shRNA) transgene, which silences VEGF-A. The aim was to find the critical level of VEGF-A reduction for vascular well-being *in vivo*. *In vitro*, the dox-inducible lentiviral shRNA vector decreased VEGF-A expression efficiently and dose-dependently in mouse endothelial cells and cardiomyocytes. In the generated transgenic mice plasma VEGF-A levels decreased shortly after the dox treatment but returned back to normal after two weeks. VEGF-A expression decreased shortly after the dox treatment only in some tissues. Surprisingly, increasing the dox exposure time and dose led to elevated VEGF-A expression in some tissues of both wildtype and knockdown mice, suggesting that dox itself has an effect on VEGF-A expression. When the effect of dox on VEGF-A levels was further tested in naïve/non-transduced cells, the dox administration led to a decreased VEGF-A expression in endothelial cells but to an increased expression in cardiomyocytes. In conclusion, the VEGF-A knockdown was achieved in a dox-regulatable fashion with a VEGF-A shRNA vector *in vitro*, but not in the knockdown mouse model *in vivo*. Dox itself was found to regulate VEGF-A expression explaining the unexpected results in mice. The effect of dox on VEGF-A levels might at least partly explain its previously reported beneficial effects on myocardial and brain ischemia. Also, this effect on VEGF-A should be taken into account in all studies using dox-regulated vectors.

## Introduction

Vascular endothelial growth factor-A (VEGF-A) is the master regulator of angiogenesis. It is effectively up-regulated in hypoxia and it binds to VEGF-receptor 1 and VEGF-receptor 2, the latter of which is the most important receptor mediating angiogenesis. In addition to promoting growth it acts as a survival factor for vascular endothelial cells and is a strong enhancer of vascular permeability [1–4]. VEGF-A has also a fundamental role in the development of vasculature, which is emphasized by the fact that the deletion of even a single allele results in early embryonic lethality due to severe vascular defects [5,6].

In adults, VEGF-A is expressed in all vascularized tissues suggesting that low physiological levels of VEGF-A are needed for the maintenance of general vascular homeostasis [7,8]. Lack of sufficient VEGF-A results in endothelial dysfunction, hypertension, ischemia and blood clotting [4,8–10]. Moreover, decrease in VEGF-A levels has been shown to cause severe thromboembolic complications, including myocardial infarctions, in up to 5% of cancer patients treated with anti-VEGF-A antibody (Avastin) [11,12]. Physiological levels of VEGF-A maintain vascular homeostasis and protection while higher levels induce physiological vascular growth. Very high levels of VEGF-A promote aberrant vascular growth with significant tissue edema [4,8].

RNA interference (RNAi) is a powerful method to silence gene expression [13]. Tetracycline-regulated gene expression systems enable to control time and quantity of gene silencing and they have been widely used in transgenic mouse models [14,15]. Lentiviral vectors are commonly used gene-delivery vehicles, which are able to transfect multiple cell types and they can also be used for the generation of transgenic mice [16, 17].

To better understand the role of VEGF-A in the adult vasculature, a VEGF-A knockdown mouse model was generated via lentiviral transgenesis [16]. In this model the mice carried a dox-regulatable VEGF-A silencing shRNA as a transgene enabling the knockdown of VEGF-A in response to dox treatment. With the mouse model the aim was to find the critical level of VEGF-A reduction for normal vascular function and to explore the pathological changes in tissues in relation to various degrees of VEGF-A silencing.

VEGF-A knockdown with the dox-induced VEGF-A shRNA was efficient *in vitro* but had only a transient and partial effect *in vivo*. Surprisingly, we found that dox itself actually regulates VEGF-A expression which could explain its previously described beneficial effects on myocardial and brain ischemia [18–22].

## Materials and methods

### Generation of VEGF-A -miR-shRNA lentiviral vector

**Fig 1:**
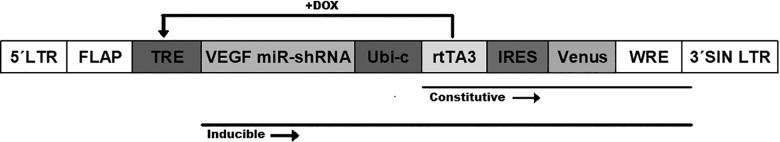
Schematic drawing of the dox-regulatable VEGF-A shRNA lentivirus vector. Lentivirus vector for inducible knockdown permits constitutive expression of a Tet-transactivating component (rtTA3) with a Venus selection marker (green fluorescent protein -like protein) and tetracycline-regulated expression of VEGF-A-shRNAs. The shRNA transcripts were designed as primary microRNA mimics i.e. they were embedded in the primary transcript of human miRNA30. The lentiviruses were self-inactivating (SIN) third generation vectors, in which part of the viral 3´LTR has been deleted preventing the viral replication. The vectors contain a central polypurine tract (indicated as FLAP) for enhancement of viral titers and a woodchuck hepatitis virus posttranscriptional regulatory element (WRE) for better transgene expression [24].

For finding the most efficient shRNA for VEGF-A knockdown, three different microRNA (miR) -shRNA constructs targeting VEGF-A were cloned into the entry vector having either normal TRE promoter (T) or tight TRE promoter (TT). The entry vectors were subcloned by site-specific Gateway recombination [23] to the parent lentivirus vector to generate the final VEGF-A -miR-shRNA expressing lentiviral vector [24]. Three shRNA sequences (sh1-sh3) targeting VEGF-A were tested. The target sequences were 5´-ATGTGAATGCAGACCAAAGAA -3`for sh1 [25–27] and 5´- AACGATGAAGCCCTGGAGTGC -3`for sh2 [28,29]. The third shRNA (sh3) sequence was searched from the RNAi codex database [30] and its target sequence was 5´- CGAGATATTCCGTAGTACATAT -3´. The control vector had a shRNA sequence targeting luciferase [24] (Fig 1).

### Lentivirus production

Third generation lentiviruses were produced by transfecting HEK293T cells with the cloned VEGF-A-shRNA plasmids using calcium phosphate transfection method as described [31]. Viral titers were measured by analyzing the amount of HIV p24 Gag antigen by ELISA and by analyzing the Venus positive viral particles with flow cytometry [31]. The p24 titers were on the order of 1x10^9^ TU/ml and the functional titers by flow cytometry analysis were on the order of 1x10^6^ vp/ml without dox and 1x10^7^ vp/ml with the dox treatment. In *in vitro* experiments the functional titers determined with dox were used for calculations.

### Testing the functionality of doxycycline-regulated VEGF-A knockdown vectors

**Fig 2:**
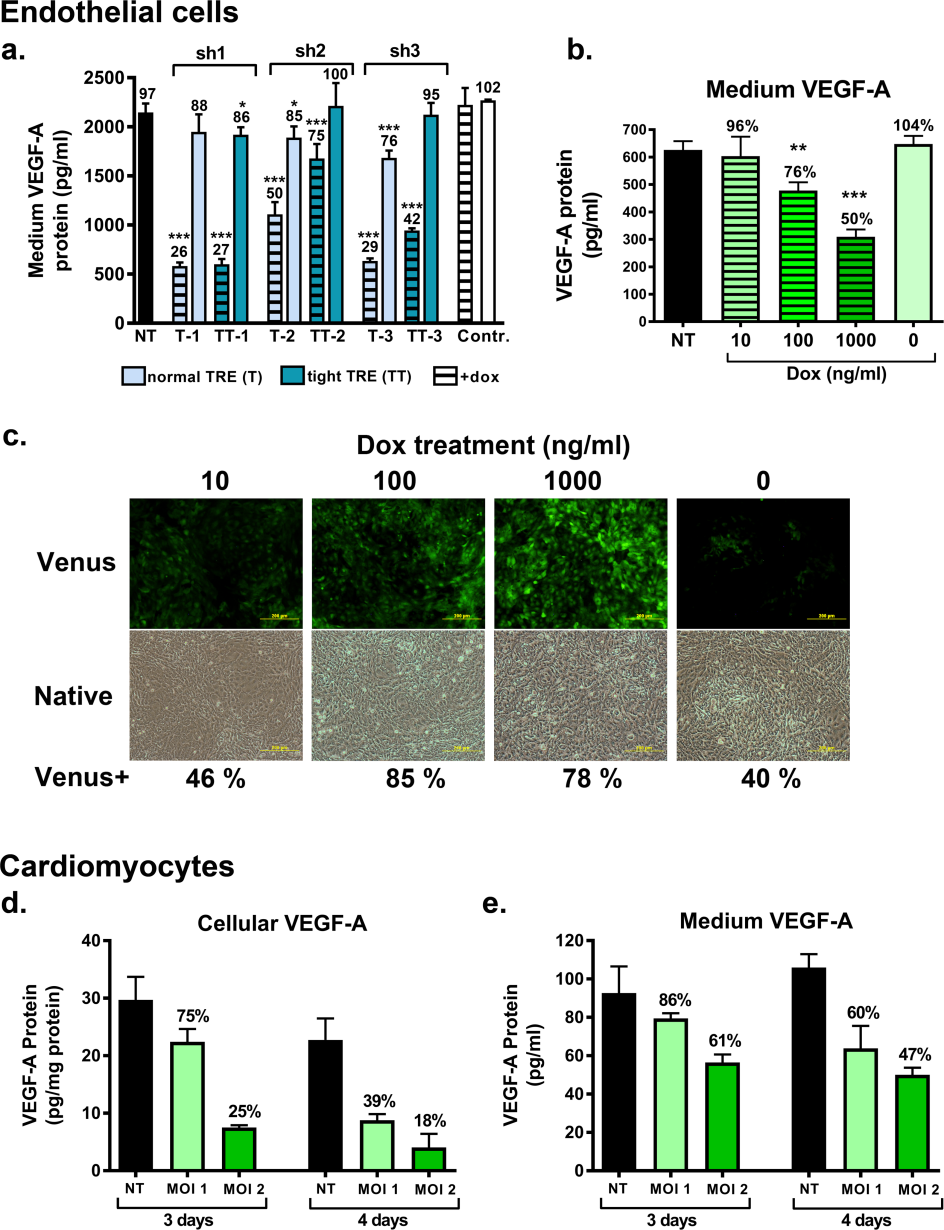
VEGF-A knockdown via RNAi in mouse endothelial cells and cardiomyocytes in a doxycycline-regulatable fashion. Secreted VEGF-A protein amount was most efficiently decreased with sh1 -knockdown vector (T-1 and TT-1) in mouse endothelial cells in comparison to sh2 (T-2 and TT-2) and sh3 (T-3 and TT-3) vectors. With the sh1 construct the normal TRE promoter (T-1) seemed to be slightly more efficient and less leaky than the tight TRE promoter (TT-1) (a). The magnitude of VEGF-A knockdown with the selected T-1 vector increased with increasing dox doses in endothelial cells (b). With the increasing dox doses also the amount of Venus expressing cells (%) was increased as follows: 40 ± 7% (–dox), 46 ± 8% (dox 10 ng/ml), 85 ± 3% (dox 100 ng/ml) and 78 ± 2% (dox 1000 ng/ml) (c). In cardiomyocytes the knockdown of VEGF-A with the T-1 vector was shown to be dependent of the amount of virus (MOI) and dox exposure time and the decrease was larger at the cellular level in comparison to the secreted protein (d-e). Results are shown as mean ± S.D., n = 3/group. The percentage of VEGF-A protein concentration compared to control +dox group (a) or non-transduced (NT) group (b, d, e) are shown above bars. *p<0.05, **p<0.01 and ***p<0.001 compared to control +dox group (a) or non-transduced (NT) group (b, d, e), 1-way ANOVA with Dunnett´s post hoc test. NT = non-transduced cells, MOI = multiplicity of infection, Contr = Control lentivirus vector targeting Luciferase. Scale bar 200 μm (c).

The VEGF-A knockdown efficiency of the six VEGF-A -shRNA expressing lentiviral vectors (T-1, TT-1, T-2, TT-2, T-3, TT-3 representing sh1, sh2 and sh3 sequences with either T or TT promoter) were tested in C166 mouse endothelial cells (American Type Culture Collection, ATCC, Manassas, VA, USA). Cells were seeded on 6-well cell culture plates and cultured in Dulbecco’s modified Eagle’s medium (DMEM) containing 10% fetal bovine serum (FBS), 1% penicillin-streptomycin and 0.584 mg/ml glutamine. One day after seeding fresh medium was changed and transductions were done by adding lentiviruses to the media with multiplicity of infection (MOI) of 1–2. On the following day fresh culture medium was added and induction started with dox concentration of 1000 ng/ml for 5 days. For non-induced cells dox was not added. In addition, non-transduced (NT) cells without viral transduction were included (Fig 2A).

The T-1 lentivirus vector was found to be the most efficient and chosen for the follow-up experiments. The dox dose response was studied with T-1 vector with MOI 4.5 and dox concentrations of 10, 100 or 1000 ng/ml for 4 days in C166 endothelial cells. When harvested the cells were monitored under microscope with phase contrast and fluorescence (Olympus IX71, Cornell University, Ithaca, NY, USA). The media was collected for VEGF-A ELISA analysis (mouse VEGF-A ELISA kit, R&D Systems, Minneapolis, MN, USA). Cells were washed with PBS and trypsinized. Trypsin was inactivated with FBS, cells were suspended and moved to Eppendorf tubes for FACS analysis and protein samples. For FACS samples the cell pellet was suspended in the fixing solution (1% PFA-1% FBS in PBS) and analyzed by FACS for Venus-positive cells. The cell pellet was lysed with western blot lysis buffer (50 mM Tris-Hcl, ph 7.5, 150 mM NaCl, 1 mM EDTA, 1% Triton X-100, 0.5% Na-deoxycholate, 0.1% SDS, 10% glycerol) with proteinase inhibitor cocktail (Halt proteinase inhibitor cocktail kit, Thermo Fisher Scientific, Waltham, MA, USA) and stored at -20°C. VEGF-A protein levels were determined with ELISA (mouse VEGF-A ELISA kit, R&D Systems, Minneapolis, MN, USA) and protein concentrations were measured with BCA Protein Assay Kit (Pierce, Rockford, IL, USA).

The effect of T-1 VEGF-A knockdown vector was also studied in HL-1 cardiomyocyte cell line cultured in Claycomb medium (Sigma-Aldrich, St Louis, MO, USA) supplied with 10% FBS– 100 μg/ml penicillin-streptomycin– 0.1 mM norepinephrine– 2 mM L-glutamine (Sigma-Aldrich, St Louis, MO, USA) with viral doses of MOI 1 and 2 and dox induction times of 3–4 days as described above. Cells were lysed by pipetting cold western blot lysis buffer T-PER Tissue Protein Extraction Reagent with Halt proteinase inhibitor cocktail (Thermo Fisher Scientific, Waltham, MA, USA) on top of the cells, after which the cells were scraped, suspended and stored at -20°C degrees.

### Dox induction studies for naïve/non-transduced cells

The effect of plain dox on VEGF-A levels was studied *in vitro*. VEGF-A mRNA expression and protein levels were studied in C166 endothelial cells and HL-1 cardiomyocytes after dox treatment with similar cell culturing conditions as described above. Depending on the timepoint studied dox was added at 24, 48 or 72h after seeding with three different concentrations of 10, 100 or 1000 ng/ml. Medium samples were collected and cells harvested 4 days after seeding as described above. In addition, for RNA isolation separate samples were prepared and the cells were lysed with TRI reagent (Sigma-Aldrich, St Louis, MO, USA) and RNA samples were further processed as described earlier [32]. Relative mRNA expression of genes were measured using specific Assays-on-Demand systems (Thermo Scientific, Waltham, MA, USA) and expression levels were normalized to peptidylprolyl isomerase A (PPIA).

### Lentiviral transgenesis

For producing transgenic VEGF-A knockdown mice, purified non-diluted T-1 lentiviruses were injected into perivitelline space of fertilized oocytes of C57Bl/6J mice (Harlan Laboratories, Indianapolis, IN) as described [16]. Mice were genotyped by PCR using designed primers: forward primer 5’- GAGCAAGTCATAAACGGCGC -3’ and reverse primer 3’- CGCGATGTGAGAGGAGAGCA -5’. The copy number of the knockdown transgene in the born transgenic mice was determined from genomic DNA isolated from kidney samples with UltraRapid Lentiviral Global Titer Kit (SBI, System Biosciences, Palo Alto, USA).

### In vivo experiments with dox treatments

A total of 109 male and female mice were included in the experiments. The VEGF-A knockdown mice (TG) and WT mice (littermates and C57Bl/6J controls (Harlan Laboratories, Indianapolis, IN)) were subjected to dox treatments for 2–10 weeks in duration. In three experiments lasting for 2, 4 and 10 weeks a dox dose of 1 mg/ml in drinking water was used and in the 5 weeks experiment the dose was doubled to 2 mg/ml. Solutions containing 1 mg/ml or 2 mg/ml doxycycline hyclate (Sigma-Aldrich, St Louis, MO, USA) and 1% (w/v) sucrose were prepared into tap water every second day and protected from light during experiments.

In 4 and 10 weeks experiments F1 progeny (6–7 months of age) were used, in 2 weeks experiment founder mice (22 months of age) were used and in 5 weeks experiment with the double dox dose F1 progeny (21–24 months of age) were used. Mice were kept in standard housing conditions in the National Laboratory Animal Centre of University of Eastern Finland, Kuopio Campus. Diet and water were provided ad libitum. At the end of the experiments mice were euthanized by carbon dioxide inhalation.

All animal studies were approved by The National Animal Experiment Board of Finland and carried out according to guidelines of The Finnish Act on Animal Experimentation. The animal experiments conformed to the Guide for the Care and Use of Laboratory Animals published by the US National Institutes of Health (NIH Publication No. 85–23, revised 1996).

### Protein and gene expression analysis

VEGF-A protein levels were determined from frozen tissue and plasma samples with mouse VEGF-A ELISA kit (R&D Systems, Minneapolis, USA) as previously described [33]. Tissue protein concentrations were analyzed using Pierce BCA assay (Thermo Scientific, Waltham, MA, USA). Relative mRNA expression of genes was measured with quantitative PCR as previously described [32].

### Statistical analyses

Results are presented as means ± SD. Statistical significances were analyzed using Student’s paired t-test when comparing two groups or one-way analysis of variance with Dunnett’s post hoc test when comparing multiple groups. The used tests are indicated in the figure legends and the following symbols are used for statistical significance: *p < 0.05, ** *p* < 0.01, *** *p* < 0.001.

## Results

### VEGF-A knockdown *in vitro* with dox-regulatable VEGF-A shRNA lentiviral vector

To find the most effective shRNA for VEGF-A downregulation for producing the VEGF-A knockdown mouse strain, three different lentiviral shRNA constructs (sh1-sh3) targeting VEGF-A with either normal (T) or tight (TT) TRE promoter were generated. The vector backbone had constitutively driven Venus fluorescent marker gene (Fig 1). The vectors were tested for their VEGF-A knockdown capacity *in vitro* in mouse endothelial C166 cell line. The sh1 construct with a normal TRE promoter (T-1) proved to be the most efficient of the tested constructs, since in response to dox treatment it depleted the secreted VEGF-A protein level to 26% when compared to control cells transduced with a control vector and treated with dox (Fig 2A). The percentage of the transduced cells was about 76%. The sh2 and sh3 constructs were less effective in decreasing the VEGF-A protein levels (Fig 2A). In the absence of dox induction, the secreted VEGF-A levels were only slightly decreased in T-1 transduced cells indicating a minimal leakage of the VEGF-A silencing vector expression cassette (Fig 2A). The T-1 construct was chosen for the further studies and for the production of the knockdown mice, since it was the most efficient in depleting VEGF-A protein levels and the leakage of the shRNA construct was minimal.

The VEGF-A knockdown with the chosen VEGF-A T-1 lentivirus vector in endothelial cells was proportional to the dox dose (Fig 2B). In addition, the number of Venus-positive cells increased in response to the dox treatment dose-dependently (Fig 2C) indicating, that the dox treatment increased the expression of constitutively driven Venus marker gene.

The T-1 vector was efficient in decreasing the cellular and secreted VEGF-A protein levels also in HL-1 cardiomyocytes (Fig 2D and 2E, respectively). The decrease in VEGF-A protein amount was larger with the higher multiplicity of infection (MOI 2 vs. MOI 1) and with a longer dox exposure time (4 days vs. 3 days) (Fig 2D and 2E). The VEGF-A knockdown was more efficient at the cellular level (Fig 2D) than at the level of the secreted protein (Fig 2E).

### Generation of VEGF-A knockdown mice

VEGF-A knockdown mice were produced via lentiviral transgenesis with the T-1 VEGF-A shRNA, which was chosen based on the *in vitro* studies. On an average 70% of the born founders (F0) were TG positive. All TG mice were born healthy with no apparent phenotype. The F0 mice were further paired and analyzed to generation F3. The transgene was transmitted through the germ line. The VEGF-A knockdown transgene copy numbers in the F0 mice were 3.0±2.2 (n = 14), the highest transgene copy number being 7. Lower copy numbers were detected in F1 progeny; in F1 pups produced by F0 x F0 breeding the copy number was 1.9±1.2 (n = 25) and in F1 progeny produced by F0 x WT breeding the copy number was 1.7±1.3 (n = 81).

### *In vivo* experiments with dox treatments

The VEGF-A knockdown mice and WT controls were subjected to dox treatments either with a dox dose of 1 mg/ml in drinking water (2, 4 and 10 weeks) or a double dose of 2 mg/ml (5 weeks). For controlling the amount of dox received during the experiments, the consumption of drinking water was measured. The amount of water mice drunk was on average 4 ml so the amount of dox consumed was on an average 4 mg/day/mouse (160 mg/kg/day).

**Fig 3:**
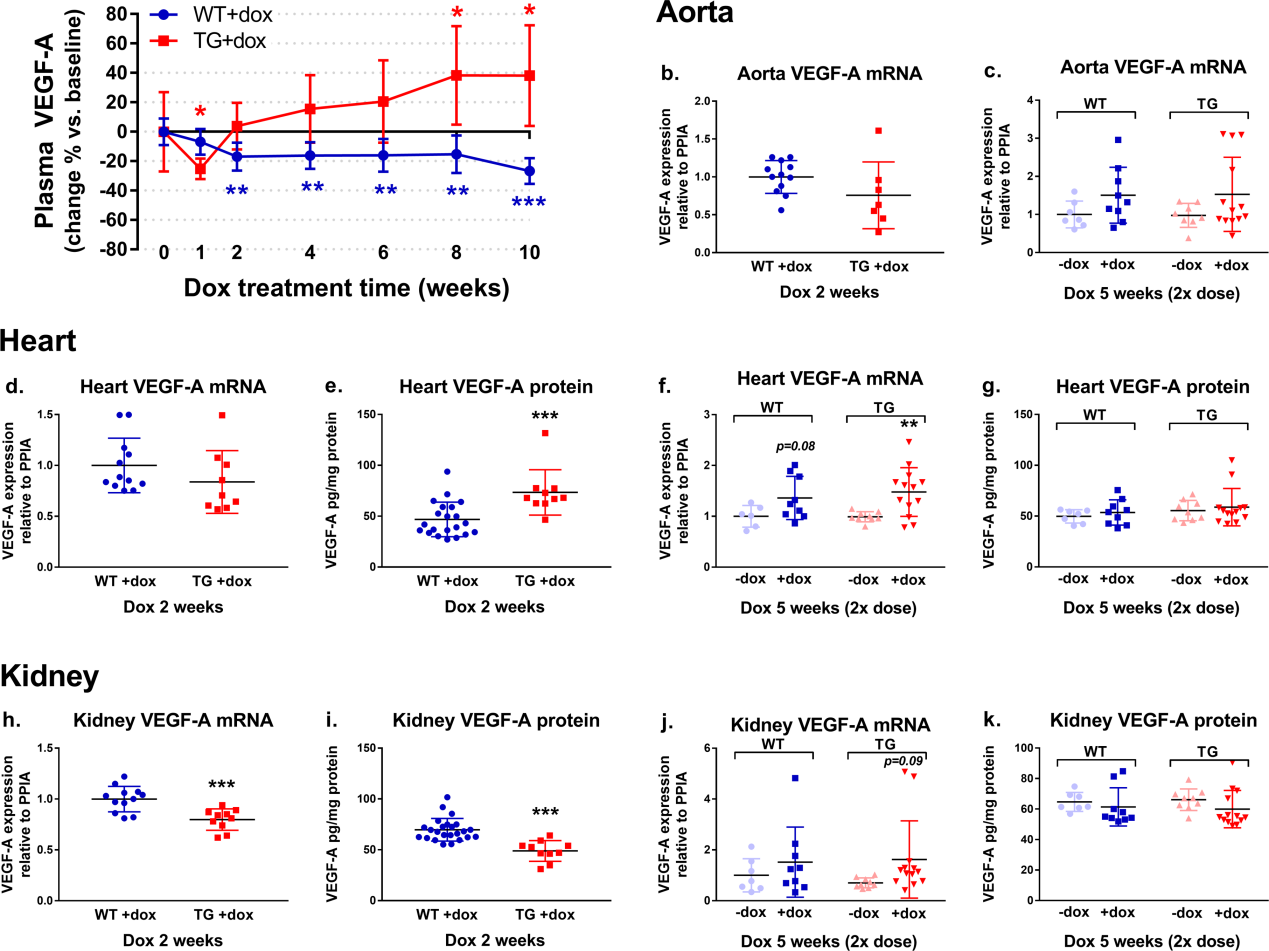
**Plasma VEGF-A concentration in response to dox treatment (a). Tissue VEGF-A mRNA and protein levels after two weeks of 1 mg/ml dox treatment and five weeks of 2 mg/ml dox dose treatment (b-k).** Dox treatment decreased plasma VEGF-A levels in TG mice after 1 week, after which the plasma VEGF-A increased. In WT mice a decreasing effect on plasma VEGF-A was seen (a). *p<0.05, **p<0.01 and ***p<0.001 compared to baseline within each group, 1-way ANOVA with Dunnett´s post hoc test, n = 9-10/group. In the selected tissues, aorta, heart and kidney, the dox treatment with the 1 mg/ml dox dose for 2 weeks showed decreasing trend in VEGF-A mRNA expression in TG mice in comparison to WT mice (b, d, h), which was associated with increased cardiac VEGF-A protein (e) and decreased kidney VEGF-A protein levels (i). When the dox dose was doubled and the treatment time increased to 5 weeks, a trend towards increasing VEGF-A expression was seen in all three tissues in both WT and TG mice (c, f, j). However, no changes were detected in protein levels (g, k). **p<0.01 and ***p<0.001 compared to WT+dox group in 2 weeks experiment (b, d, e, h, i) or to no dox group (-dox) in 5 weeks experiment (c, f, g, j, k), *t-test*, n = 6-24/group.

The plasma VEGF-A protein level measured from tail vein blood samples in TG mice in response to dox treatment (1 mg/ml) initially decreased after one week of dox exposure, where after it started to rise towards the 10 week time point (Fig 3A). Instead, in WT mice, the plasma VEGF-A levels declined throughout the 10 week follow-up (Fig 3A).

After 2 weeks of dox treatment (1 mg/ml) VEGF-A mRNA expression was reduced in kidney (Fig 3H) and a trend towards a decreased mRNA level was also seen in aorta (Fig 3B) and heart (Fig 3D) of TG mice in comparison to WT mice. VEGF-A protein amount was decreased in the kidney (Fig 3I) but in contrast increased in the heart (Fig 3E) 2 weeks after the dox treatment in TG mice.

After 4 weeks, VEGF-A mRNA expression was somewhat decreased in all studied tissues (heart, kidney, lungs, spleen, skeletal muscle, liver) of TG mice in comparison to WT mice in response to dox treatment, although the changes were significant only in the lungs and spleen (data not shown). However, 10 weeks after the dox treatment the VEGF-A mRNA levels showed an increasing trend in all studied tissues (heart, kidney, lungs, spleen, skeletal muscle, liver) in TG mice in comparison to TG mice without the dox treatment (data not shown).

Dox treatment with the double dox dose (2 mg/ml) for 5 weeks led to significantly increased VEGF-A mRNA expression in the heart of TG mice (Fig 3F) and tended to have an increasing effect on the VEGF-A mRNA levels in the heart of WT mice (Fig 3F) and also in the aorta (Fig 3C) and kidney (Fig 3J) of both TG and WT mice when compared to no dox controls. The VEGF-A protein levels in heart (Fig 3G) and kidney (Fig 3K) remained unchanged.

**Fig 4:**
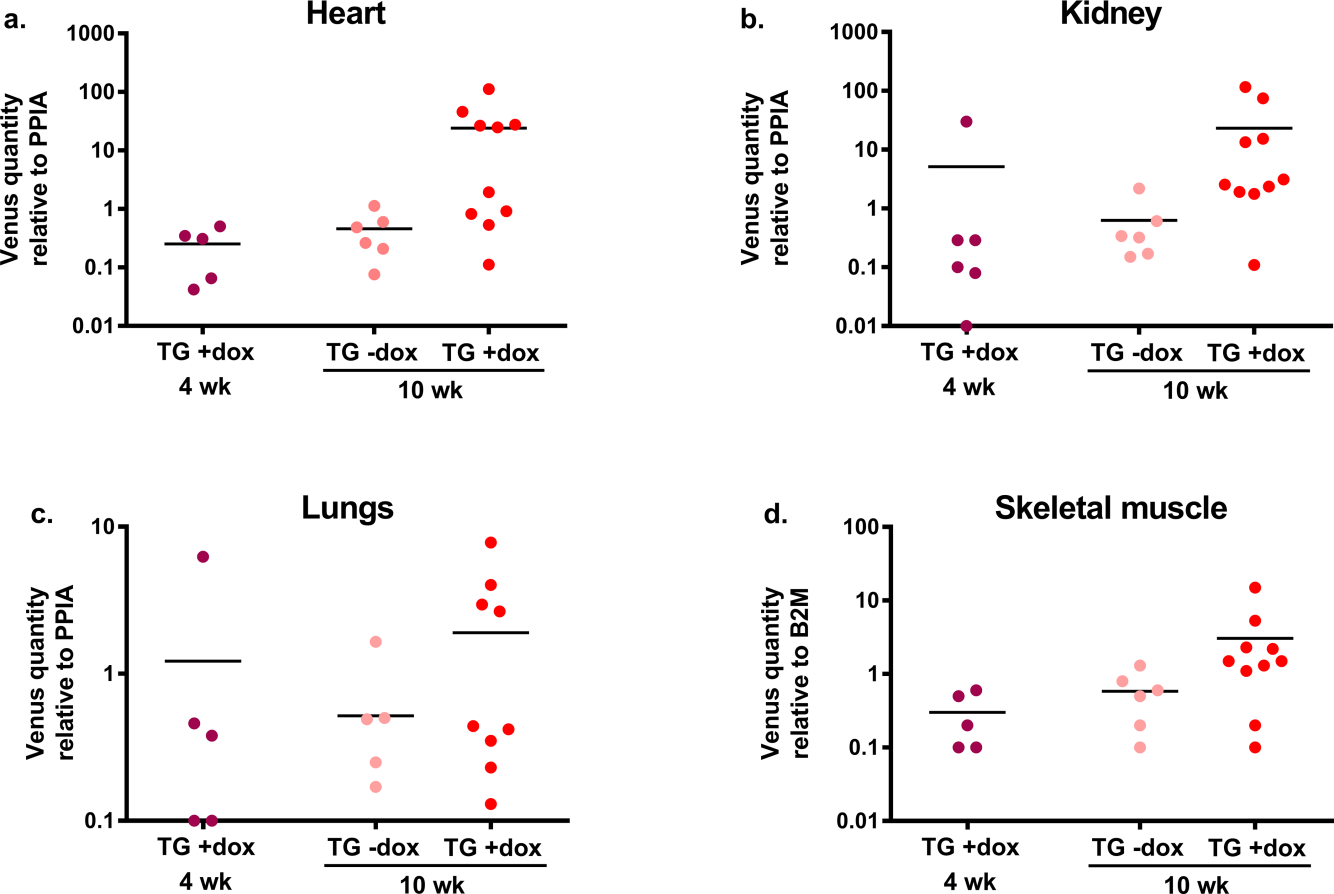
**Tissue Venus mRNA expression after 4 and 10 weeks of dox treatment in VEGF-A knockdown TG mice (a-d).** Dox treatment increased the Venus expression in the heart (a), kidney (b), lungs (c) and skeletal muscle (d) in TG mice after 10 weeks of dox treatment in comparison to TG mice without dox treatment. The Venus mRNA expression was generally higher after 10 weeks dox treatment when compared to 4 weeks dox treatment in TG mice (a-d). No Venus expression was detected in the WT mice with 4 weeks dox treatment (data not shown). n = 5-10/group.

Similarly with the *in vitro* experiments, the basal Venus expression was increased in TG mice in response to dox (1 mg/ml) treatment. The increase in Venus mRNA level was dependent on the studied tissue (heart, kidney, lungs, spleen, skeletal muscle) as it varied from 1.2-fold increase in spleen (data not shown) to 52-fold increase in heart and was on an average 20-fold higher in TG mice receiving dox for 10 weeks in comparison to TG mice without dox (Fig 4A–4D). In addition, Venus expression increased along with the dox exposure time, being about 23-fold higher after 10 weeks of dox (1 mg/ml) as compared to 4 weeks of dox in TG mice varying from 1.5-fold increase in spleen (data not shown) to 95-fold increase in heart (Fig 4A). The WT mice treated with 4 weeks of dox showed no Venus expression (data not shown).

### The effect of dox treatment on VEGF-A expression *in vitro*

**Fig 5:**
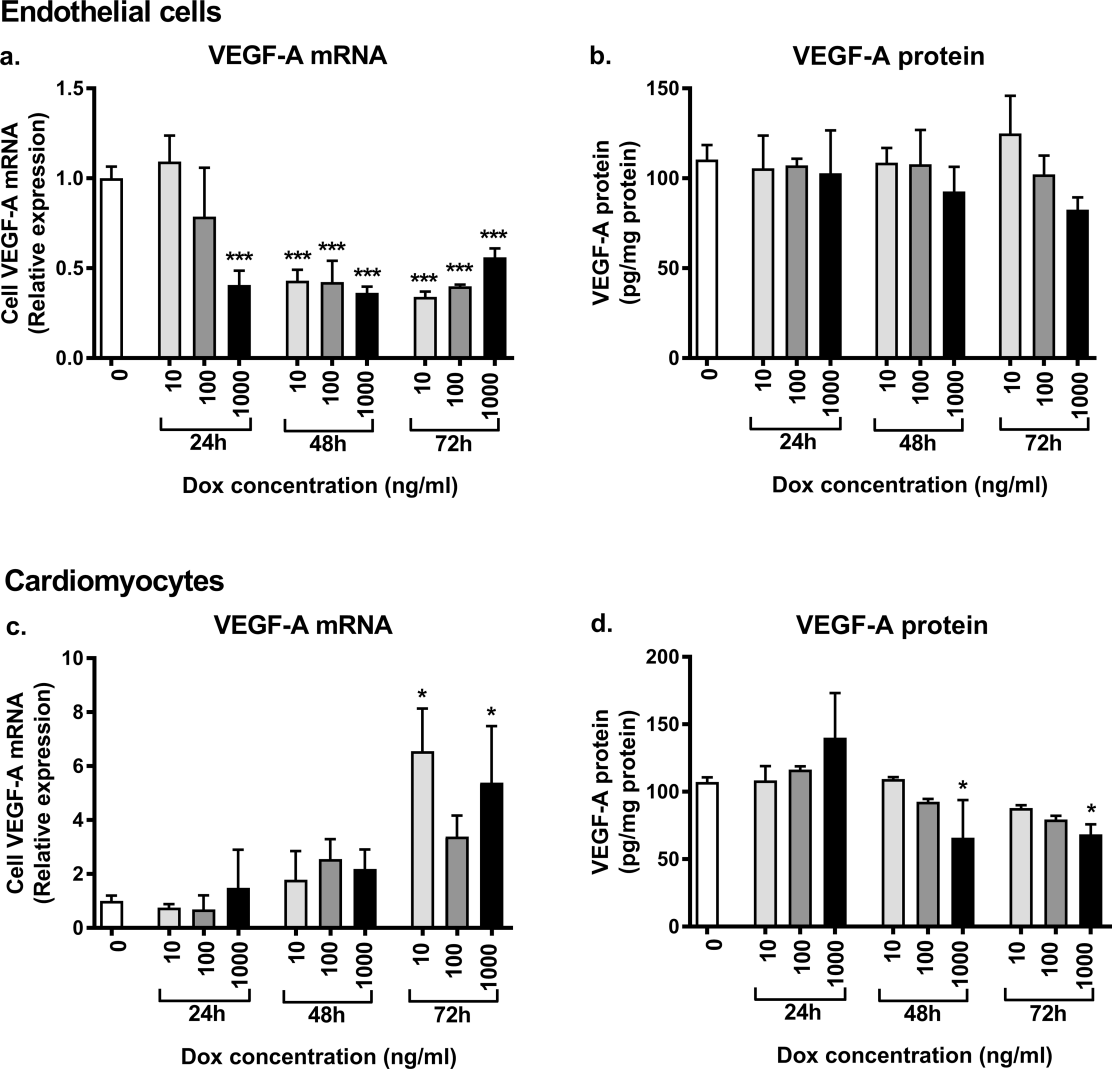
**VEGF mRNA and protein levels in naïve/non-transduced mouse endothelial cells (a-b) and cardiomyocytes (c-d) *in vitro* after dox treatment of 10–1000 ng/ml for 24–72 h.** VEGF-A mRNA levels were significantly decreased in endothelial cells 48–72 h after dox treatment with all used dox doses and already at 24 h with the highest dose of 1000 ng/ml (a). The cellular VEGF-A protein levels remained unchanged (b). In cardiomyocytes the VEGF-A mRNA levels increased (c) and cellular VEGF-A protein amount decreased after dox treatment of 48–72 h (d). Results are shown as mean ± S.D., one-way ANOVA with Dunnett’s *post hoc* test, *p<0.05, ***p<0.001 compared with the group of no dox treatment (0 ng/ml), n = 3/group.

As it was noted with *in vivo* experiments, that dox itself seemed to regulate VEGF-A expression, the effect of plain dox treatment, independently of the dox-regulatable transgene, on VEGF-A expression was further studied *in vitro* in naïve/non-transduced cells. In endothelial cells, the VEGF-A mRNA levels were markedly decreased 48–72 h after the dox treatment with all used dox doses and already at 24 h with the highest dose of 1000 ng/ml (Fig 5A). Cellular VEGF-A protein levels were not significantly changed (Fig 5B). In cardiomyocytes the VEGF-A mRNA levels increased (Fig 5C) and cellular VEGF-A protein amount decreased (Fig 5D), the change being most prominent at 72 h timepoint and with the largest dox dose.

## Discussion

VEGF-A is the most important growth factor affecting the vasculature and its growth [1–3]. It is the primary target for several pro-angiogenic therapies for treating ischemia [4] and also for anti-angiogenic therapies for treating cancer and age-related macular degeneration [2,12,34]. VEGF-A is expressed in virtually all vascularized tissues [7] and it is essential for the maintenance of vascular well-being; neutralizing circulating VEGF-A leads to endothelial dysfunction and high blood pressure in animals [9] and to serious thromboembolic complications like myocardial infarction, stroke or deep vein thrombosis in humans [11,12]. Although the essential role of VEGF-A is well established in vascular growth, its role in mature vasculature is not well understood.

For studying the role of VEGF-A in the adult, we developed a new transgenic mouse model with conditional VEGF-A knockdown. Knockdown transgenesis suited well for our purpose, since it is a valuable method to investigate genes that are lethal when knocked out completely, as is the case with VEGF-A [5,6]. Lentiviral perivitelline-injection method was used for the transgenesis, since it is an effective and safe method to produce knockdown mice and has clear advantages over the traditional plasmid microinjection method [16]. The cloned lentiviral vector included Tet-On system -driven shRNA targeting VEGF-A, which enables inducible knockdown of VEGF-A with well tolerated antibiotic doxycycline (dox) and constitutive expression of the fluorescent marker protein Venus. By inducing the knockdown of VEGF-A expression in this mouse model, we initially aimed to determine the critical VEGF-A level for normal physiological homeostasis and the level where pathological changes begin to occur.

Prior to creating the mouse model, the efficacy of the cloned dox-inducible VEGF-A knockdown lentivirus vector was tested *in vitro* in endothelial cells and cardiomyocytes. The lentiviral vector effectively silenced VEGF-A expression in response to dox treatment *in vitro* leading up to 82% decrease in cellular VEGF-A protein in cardiomyocytes. No leaky expression of shRNA in the absence of dox induction was seen. VEGF-A knockdown was shown to be dox dose-dependent and the constitutive Venus expression was increased in response to dox treatment, as described previously [24].

For the VEGF-A knockdown mice on average 70% of the born founder mice were transgenic. Similar good lentiviral transgenesis rates have been achieved in our group also previously [16,35]. When producing transgenic mice via lentiviral transgenesis, each born mouse is unique regarding the transgene copy number and genome insertion point [16,36]. In the produced knockdown mice the transgene copy number was ≈ 3 in F0 generation and declined in F1 generation to ≈ 2 copies on average, which is in line with previous reports on the lentivirally produced transgenic mice [35,37].

The major factor determining the level of target gene silencing is the knockdown transgene copy number [36]. In addition, in conditional knockdown models, the dosage of gene inducing agent plays a major role. Dox is a well-tolerated tetracycline derivate widely used in clinics and in conditional animal models, which allows researchers to control duration and degree of gene silencing [14]. In this study, the mice drank the dox drinking water solution on average 4 ml/ day/ mice, which is in the normal range of mouse water consumption [38]. Depending on the animal model dox doses of 2 μg/ml to 2 mg/ml in drinking water are needed for inducing gene expression [15]. Herein the dox doses used were at the higher end of the previously described doses being 1–2 mg/ml in drinking water which was administered for 2–10 weeks. Already the dox dose of 1 mg/ml increased markedly (20-fold on average) the constitutive Venus mRNA expression in TG mice after 1 week treatment indicating that the used amount of dox was able to induce the expression of VEGF-A knockdown construct.

In the knockdown mouse model partial VEGF-A silencing was achieved after a short term dox (1 mg/ml) administration of 1 week in plasma protein level and after 2 weeks administration in e.g. kidneys at mRNA level when compared to wt controls indicating that the achieved transgene copy numbers and dox concentrations were functional. However, doubling the dox dose and increasing the dox exposure time to 5 weeks did not lead to any better knockdown but, in contrast, to increased VEGF-A mRNA levels in some tissues of both TG and WT mice in comparison to no dox controls indicating that dox itself upregulated VEGF-A mRNA expression. However, the effect of dox treatment was not consistent, since VEGF-A expression was either up- or downregulated depending on the tissue, time of dox exposure and whether mRNA or protein levels were measured. Therefore, although the VEGF-A knockdown with the lentiviral shRNA vector was efficient *in vitro*, the VEGF-A knockdown with the same vector was not achievable in the *in vivo* model.

Due to the variable effects of dox on VEGF-A expression seen in the knockdown mice, the effect of plain dox was further studied in naïve endothelial cells and cardiomyocytes *in vitro*, where the VEGF-A expression was markedly decreased in endothelial cells but increased in cardiomyocytes. The results from the knockdown mice and *in vitro* experiments with naïve cells are difficult to compare. However, the VEGF gene expression was increased both *in vivo* and *in vitro* in cardiac cells/cardiomyocytes with the higher dox doses and longer administration times. To the best of our knowledge, there are no previous reports showing that dox regulates VEGF-A expression.

Dox has been shown to have beneficial effects in ischemia models. Dox treatment decreases the infarct size after transient middle cerebral artery occlusion [21] and reduces long-term cerebral tissue loss and white matter injury after hypoxic-ischemic injury [22]. Cardioprotective effects of dox after myocardial infarction have also been shown in rats [36] and in clinical trials, where the dox treatment reduced adverse post-myocardial infarction left ventricular remodelling and also decreased infarct size [19,20].

The dox mechanism of action remains speculative. Previously it has been hypothesized that the beneficial effects in ischemia may be related to its ability to act as a scavenger of reactive oxygen species, an anti-apoptotic agent and an inhibitor of extracellular matrix degrading metalloproteinases [19,39]. In addition dox inhibits TNF-α-converting enzyme preventing TNF-α release and indirectly serine proteases, that are elevated in chronic wounds, the healing of which was improved with topical dox [40]. Furthermore, in a colon cancer study in rats it was shown that dox treatment induced Nuclear factor (NF) -ĸB activation and upregulation of VEGF-A, and it also enhanced tumorigenesis possibly by increasing tumor angiogenesis and metastasis [41]. Dox treatment can also change the composition of the gut microbiome, which in turn can alter the immune system and the expression of cytokines and transcription factors [42,43].

Dox has multiple properties in addition to antimicrobial ones, the mechanisms of which are not fully known. This should be taken into account when using dox-inducible animal models and proper controls for studying the role of plain dox should always be included in the study design. Our data demonstrates that dox regulates VEGF-A expression. The effect of dox on VEGF-A expression seemed to interfere with the effect of the VEGF-A knockdown vector in the tissues of TG mice making it very difficult to study VEGF-A biology in the dox-inducible knockdown models. The effect of dox on endogenous VEGF-A expression might provide an explanation for the beneficial effects of dox in several ischemia models, as VEGF-A regulates vascular growth. Our findings also suggest that the association between dox and VEGF-A expression should be taken into account in all future studies using the dox-inducible models *in vitro* and *in vivo*.
